# Selective effects of protein 4.1N deficiency on neuroendocrine and reproductive systems

**DOI:** 10.1038/s41598-020-73795-6

**Published:** 2020-10-12

**Authors:** Hua Wang, Marilyn Parra, John G. Conboy, Christopher D. Hillyer, Narla Mohandas, Xiuli An

**Affiliations:** 1 Red Cell Physiology Laboratory, New York Blood Center, New York, NY 10065 USA; 2 Department of Pathology, School of Basic Medical Sciences, Peking University Health Science Center, and Peking University Third Hospital, Beijing, 100191 China; 3 Biological Systems and Engineering Division, Lawrence Berkeley National Laboratory, Berkeley, CA 94720 USA; 4 Laboratory of Membrane Biology, New York Blood Center, 310 East 67th St, New York, NY 10065 USA

**Keywords:** Developmental biology, Endocrinology

## Abstract

Protein 4.1N, a member of the protein 4.1 family, is highly expressed in the brain. But its function remains to be fully defined. Using 4.1N^−/−^ mice, we explored the function of 4.1N in vivo. We show that 4.1N^−/−^ mice were born at a significantly reduced Mendelian ratio and exhibited high mortality between 3 to 5 weeks of age. Live 4.1N^−/−^ mice were smaller than 4.1N^+/+^ mice. Notably, while there were no significant differences in organ/body weight ratio for most of the organs, the testis/body and ovary/body ratio were dramatically decreased in 4.1N^−/−^ mice, demonstrating selective effects of 4.1N deficiency on the development of the reproductive systems. Histopathology of the reproductive organs showed atrophy of both testis and ovary. Specifically, in the testis there is a lack of spermatogenesis, lack of leydig cells and lack of mature sperm. Similarly, in the ovary there is a lack of follicular development and lack of corpora lutea formation, as well as lack of secretory changes in the endometrium. Examination of pituitary glands revealed that the secretory granules were significantly decreased in pituitary glands of 4.1N^−/−^ compared to 4.1N^+/+^. Moreover, while GnRH was expressed in both neuronal cell body and axons in the hypothalamus of 4.1N^+/+^ mice, it was only expressed in the cell body but not the axons of 4.1N^-/-^ mice. Our findings uncover a novel role for 4.1N in the axis of hypothalamus-pituitary gland-reproductive system.

## Introduction

Protein 4.1N^1^ is a neuronally enriched member of the protein 4.1 family which also include protein 4.1R^2^, 4.1G^3^ and 4.1B^4^ homologs. Two highly conserved domains, the 4.1-ezrin-radixin-moesin (FERM) domain and the spectrin/actin-binding domain (SABD) are responsible for interactions of 4.1 family of proteins with the plasma membrane proteins and with the spectrin–actin cytoskeleton, respectively^5^. The third conserved domain in the 4.1 family is the carboxy-terminal domain (CTD). It has been shown that all members of the protein 4.1 family undergo extensive tissue specific alternate splicing generating multiple protein 4.1 isoforms. In the case of 4.1N, the 135 kDa 4.1N is the predominant isoform identified in the brain, whereas a smaller 100 kDa isoform is enriched in peripheral tissues, such as the enteric neurons of the intestinal tract and adrenal gland^6^.

In vitro cell biological studies have identified multiple cellular functions for protein 4.1N. Anti-proliferative actions of NGF in PC12 cells has been shown to be due to binding of 4.1N to the nuclear mitotic apparatus protein (NuMA) following its translocation to the nucleus and preventing the mitotic actions of NuMA^7^. Furthermore, the binding of 4.1N to PIKE prevents its interactions with nuclear PI3K, which can influence the regulation of PI3K by NGF^8^. Interaction of protein 4.1N with dopamine receptor is required for the localization or stabilization of dopamine receptors at the neuronal plasma membrane^9^. Among dopamine receptors, protein 4.1N interacts specifically with D2 and D3 dopamine receptors. Moreover, the protein 4.1N-binding region of inositol 1,4,5-trisphosphate receptor type 1 (IP_3_R1) is necessary and sufficient for the localization of IP_3_R1 at the basolateral membrane domain in polarized MDCK cells^10^. Both the CTT14aa and CTM1 sequences of IP_3_R1 can bind to 4.1N ^11^, suggesting that the spectrin-actin binding domain of 4.1N can serve as a linker between IP_3_R1 and actin filaments. This actin filament-dependent regulation of IP_3_R1 diffusion may be important for the spatiotemporal regulation of intracellular Ca^2+^ signaling which in turn mediates neurite formation^12–14^. Interestingly, 4.1N does not interact with the IP_3_R in an epithelial cell line WIF-B, implying cell specific interaction between protein 4.1N and the IP_3_R and tissue-specific mechanism in shaping the pattern of Ca^2+^ waves in various cell types^15^. In primary hippocampal cultures, mouse 4.1N is enriched at the discrete sites of synaptic contact, co-localizing with the postsynaptic density protein of PSD95 and GluR1, suggesting a potential functional role for 4.1N as a component of the cytoskeletal architecture of excitatory synapses^16^. 4.1N also directly interacts with the GluR1 subunit of the AMPA receptor and co-localizes with AMPA receptors at excitatory synapses^17^.

In contrast to our understanding of the multiple functions of 4.1N in various cell types based on in vitro cell biological studies, function of 4.1N in vivo is yet to be defined. To address this issue, we generated 4.1N^−/−^ mice and performed phenotypic characterization of these mice to begin to decipher the function of 4.1N in vivo. The 4.1N^−/−^ mice were born at lower birth rate and showed slow growth with marked hypoplasia of reproductive system. These findings in conjunction with documentation of focal expression pattern of 4.1N in pituitary and hypothalamus suggest a specialized function for 4.1N protein in neuroendocrine system.

## Results

### Targeted disruption of the 4.1N gene

**Figure 1:**
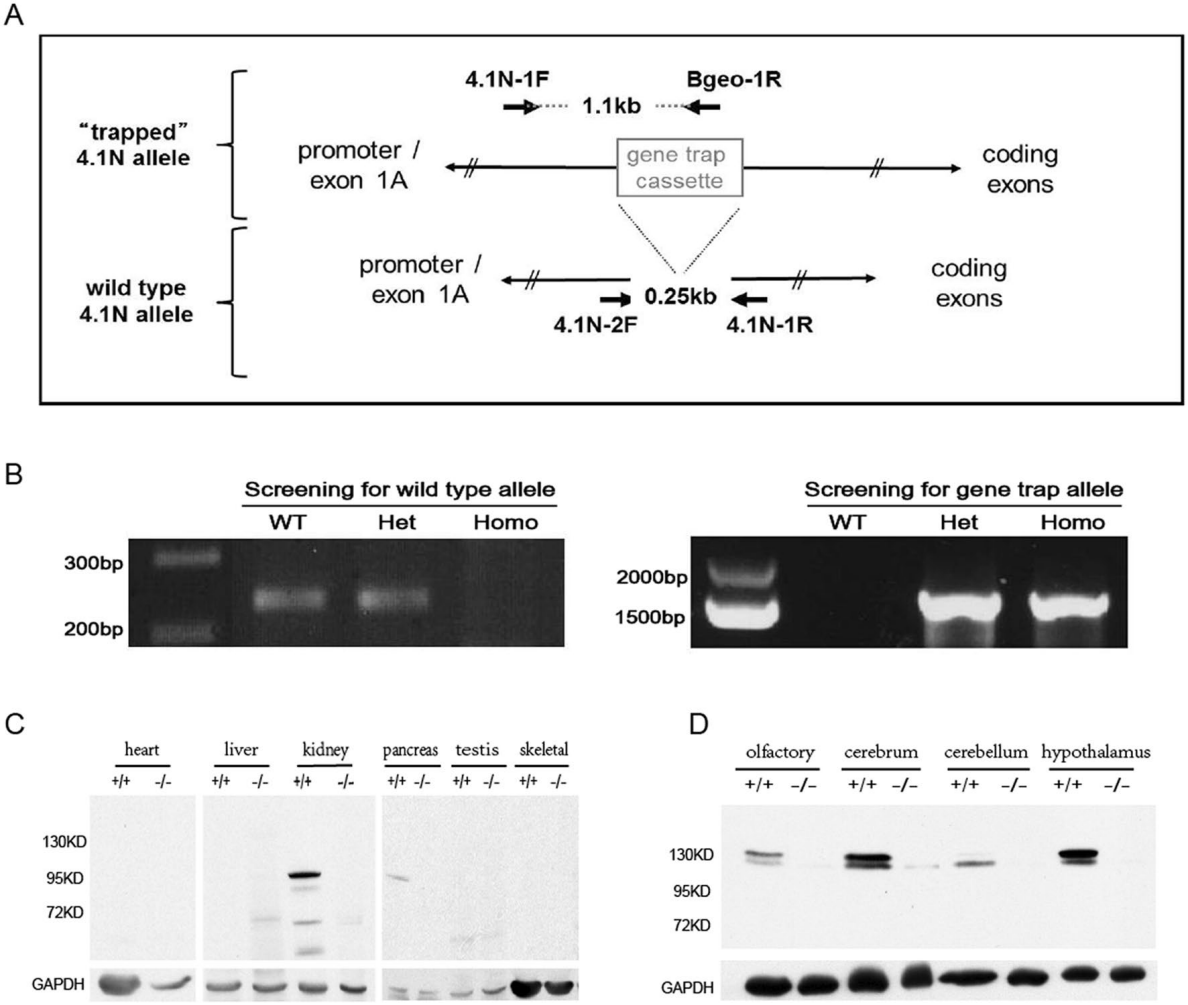
Disruption of 4.1N gene expression. **(A)** A gene trap cassette with an insertion downstream of exon 1A and upstream of exon 2 in the 4.1N gene. **(B)** Genotype analysis. Offspring of heterozygous mating pairs were screened by PCR using primer pairs that distinguish the wild type allele from the knockout allele. **(C)** Western blot analysis of multiple tissues. A prominent ~ 95 kDa band was detected in the kidney, pancreas, with a very faint lower band in the kidney, pancreas and testis. No signal was evident in the heart, skeletal muscle and liver. **(D)** Western blot analysis in various regions of brain. Two 4.1N bands are revealed with the major one migrating at ~ 135 kDa expressed in olfactory, cerebrum and hypothalamus, whereas the larger splice form of 4.1N showed low abundance in cerebellum. Both bands are absent in 4.1N^−/−^ extract. For (**C**) and (**D**), GAPDH was used as control. The full length blots for (**C**,**D**) are shown in supplementary Fig S3.

To study the roles of 4.1N in vivo, we generated 4.1N knockout mice using Embryonic stem (ES) cells generated by the International Gene Trap Consortium. A gene trap cassette with a reported insertion site between exon 1A and exon 2 was confirmed by genotype analysis using primer pairs (Fig. 1A) that could distinguish the wild type allele from the knockout allele (Fig. 1B). Western blot analysis showed that the homozygous gene trap allele reduced expression levels of 4.1N protein to undetectable levels in various tissues tested (Fig. 1C,D and Suppl. Fig S3), confirming the generation of a 4.1N null phenotype. Heterozygous mice (4.1N^+/−^) expressing ~ 50% of the normal level of 4.1N were fertile and phenotypically indistinguishable from wild type mice (supplementary Fig. S4,S5).

### Expression of 4.1N in brain and other tissues by Western blot analysis

The protein 4.1N undergoes tissue specific alternate splicing generating multiple isoforms. We compared the expression of 4.1N in different parts of brain and other tissues by Western blot analysis. Western blot analysis of hypothalamus tissue with 4.1N-specific polyclonal antibody revealed a doublet, with the predominant band migrating at 135 kDa and a less prominent lower molecular weight band. A similar pattern was seen for olfactory and cerebrum, whereas the lower molecular band was more prominent than the 135 kDa band in cerebellum (Fig. 1D). A very faint band of 165 kDa was also detected following longer exposures and may represent a larger splice form of 4.1N of low abundance. A prominent 95 kDa band was detected in the kidney and pancreas, in association with a very faint lower MW band seen in the kidney, pancreas and testis (Fig. 1C). The 95 kDa isoform likely corresponds to an alternatively spliced isoform that excludes intervening sequence between the SABD and CTD^6^. Neither the 95 kDa or 135 kDa 4.1N isoforms were found in heart, liver, skeletal muscle and testis.

### 4.1N^−/−^ mice exhibit increased mortality and movement defects

Because both male and female 4.1N^−/−^ mice were infertile, the 4.1N^−/−^ mice were obtained by breeding 4.1N^+/−^ male and female mice. While the 4.1N^+/+^ and 4.1N^+/−^ mice were obtained at the expected Mendelian ratio following breeding of heterozygous 4.1N^+/−^ mice, there was a marked decrease in the number of live born homozygous 4.1N^−/−^ mice. Among the 321 male progenies there were 108 4.1N^+/+^, 177 4.1N^+/−^ and 36 4.1N^−/−^ mice while among 305 female progeny there were 85 4.1N^+/+^, 182 4.1N^+/−^ and 38 4.1N^−/−^ mice, implying that complete 4.1N deficiency results in significant intrauterine and neonatal mortality. Furthermore, deaths were common among the surviving 4.1N^−/−^ mice: 19 of 36 (53%) of males and 16 of 38 (43%) of females died between 3 to 5 weeks of age, whereas all their wild-type and hetero-type littermates were alive at that time. These young 4.1N^−/−^ mice were marasmus, weak with movement defects, and with growth retardation. Detailed examination of the 4.1N^−/−^ mice revealed that they were passive and when gently lifted by their tail, their movements were powerless relative to those of their wild-type counterparts. When tested for their ability to walk on a rod or on top of a container, some of the 4.1N^−/−^ mice did not move at all while others were able to proceed for a short distance and tired quickly which was in marked contrast to wild type mice that were able to accomplish these tasks readily. The observed neurobehavioral defects in 4.1N^−/−^ mice suggest a potential role for 4.1N in brain function.

### Slow growth and decreased weight of reproductive organs in 4.1N^−/−^ mice

**Figure 2:**
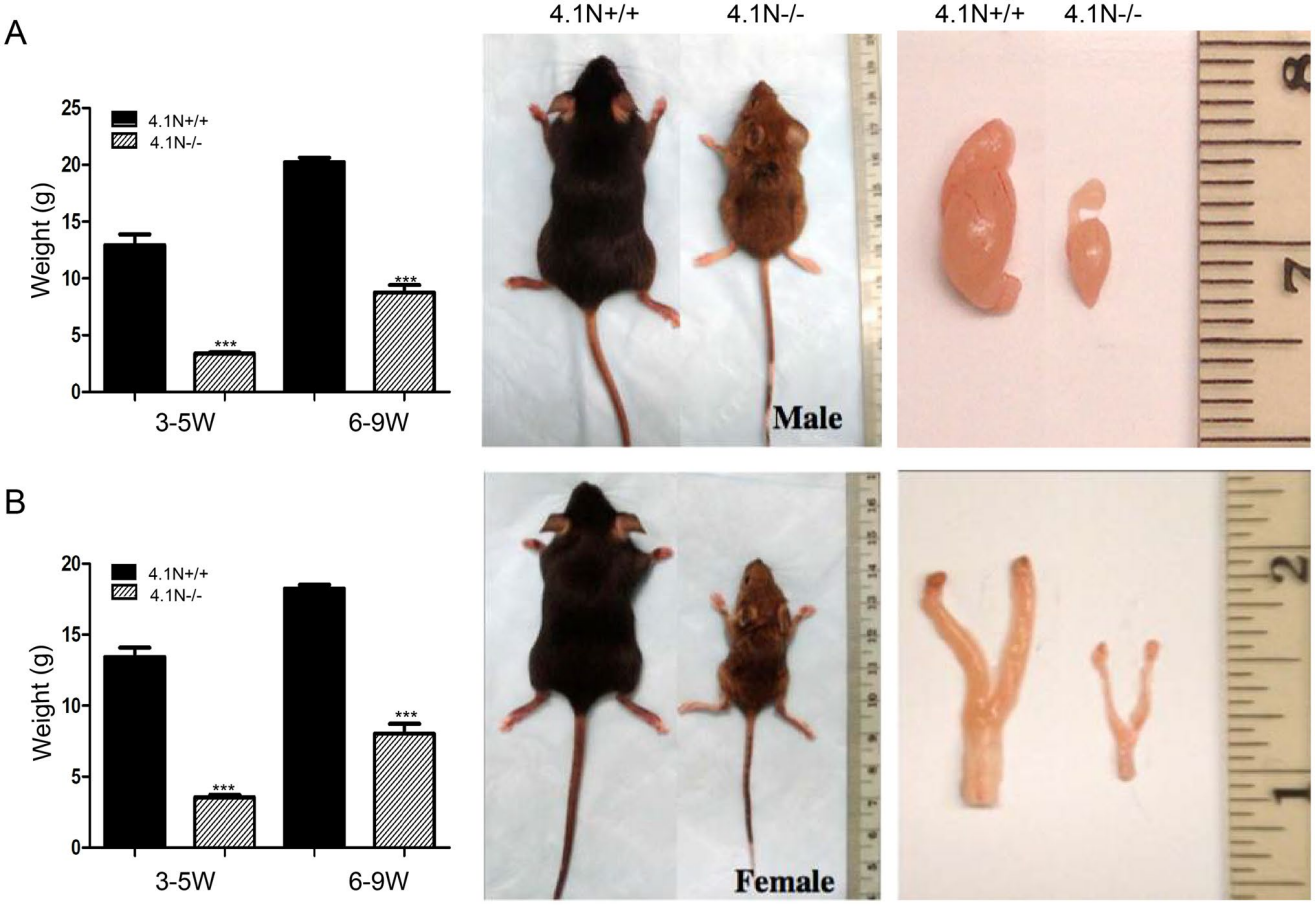
Outward appearance and gross appearance of the reproductive organs of adult mice. 4.1N^−/−^ 8-week-old male (**A**) and 6-week-old female (**B**) mice showed obvious growth retardation associated with decreased weight and size compared to 4.1N^+/+^ littermates. Bar graphs demonstrate that body weight of 4.1N-null mice was reduced compared with wild-type littermates both in males (**A**) and females (**B**) at 3–5 weeks (P < 0.001, n = 6) and 6–9 weeks (P < 0.001, n = 10). Macroscopic view of the testis and epididymis at 8 weeks of age from the control 4.1N^+/+^ littermates and 4.1N^−/−^ mice showed that the male reproductive organs are dramatically smaller in the 4.1N^−/−^ mice (**A**). Bottom panels are macroscopic views of the ovary and uterus at 20 weeks of age from the control 4.1N^+/+^ littermates (left) and 4.1N^−/−^ mice showing these organs are much smaller in the 4.1N^−/−^ mice.

As the mice aged, we noted a marked age-dependent decrease in size and body weight of 4.1N^−/−^ mice compared with wild-type littermates (Fig. 2). Body weight of 4.1N^−/−^ mice was reduced by 3 to 4 fold compared to their wild-type littermates for both males (Fig. 2A) and females (Fig. 2B) at 3–5 weeks of age (P < 0.001), and reduced by 2 to 3 fold at 6–9 weeks of age (P < 0.001, Fig. 2). We then monitored the weight of different organs in the age-matched wild-type and knockout mice (6 to 9 weeks old mice). The size and weight of the male and female reproductive organ were noticeably decreased in 4.1N^−/−^ mice compared to that of 4.1N^+/+^ (Fig. 2). A detailed tissue weight assessment showed that the ratio of weight of the testis and ovary to the body weight for 4.1N^−/−^ male and female mice was dramatically decreased compared with the 4.1N^+/+^ control mice (Table1). The 4.1N^−/−^ adult testis weight was 0.13 ± 0.018% of body weight, in contrast to 0.41 ± 0.035% of body weight (p < 0.0001) in 4.1N^+/+^ mice. For the female, the 4.1N^−/−^ adult ovary weight was 0.0073 ± 0.00085% of body weight, while it was 0.014 ± 0.0013% (p = 0.0005) in 4.1N^+/+^mice. We also noted that the 4.1N^−/−^ mice displayed a mild decrease in liver weight compared with the control wild-type littermates (p < 0.05). No significant difference for the ratio of organ to body weight was noted for other organs including heart, lung, spleen and kidney in 4.1N^−/−^ mice.

**Table 1:** Changes of organs weight in 4.1N-/- mice.

Parameters	4.1N^+/+^ (organ/body,g)	4.1N^+/+^ (% of body weight)	4.1N^-/-^ (organ/body,g)	4.1N^-/-^ (% of body weight)
Testis weight, n = 8	0.085 ± 0.0097/20.56 ± 1.11	0.41 ± 0.035	0.011 ± 0.0026/8.63 ± 1.85	0.13 ± 0.018***
Ovary weight, n = 6	0.0027 ± 0.00027/18.56 ± 0.64	0.014 ± 0.0013	0.00061 ± 0.00013/8.27 ± 1.38	0.0073 ± 0.00085***
**Liver weight, n = 6**				
Male	1.087 ± 0.067/20.65 ± 0.95	5.26 ± 0.17	0.40 ± 0.09/8.67 ± 1.92	4.63 ± 0.63*
Female	0.835 ± 0.084/18.56 ± 0.64	4.49 ± 0.38	0.328 ± 0.08/8.27 ± 1.38	3.95 ± 0.55*
**Heart weight, n = 6**				
Male	0.117 ± 0.0098/20.65 ± 0.95	0.57 ± 0.054	0.049 ± 0.0093/8.67 ± 1.92	0.56 ± 0.051
Female	0.102 ± 0.012/18.56 ± 0.64	0.55 ± 0.048	0.044 ± 0.0073/8.27 ± 1.38	0.53 ± 0.033
**Lung weight, n = 6**				
Male	0.143 ± 0.014/20.65 ± 0.95	0.69 ± 0.055	0.065 ± 0.0068/8.67 ± 1.92	0.77 ± 0.12
Female	0.155 ± 0.02/18.56 ± 0.64	0.83 ± 0.09	0.065 ± 0.01/8.27 ± 1.38	0.78 ± 0.05
**Spleen weight, n = 6**				
Male	0.064 ± 0.0086/20.65 ± 0.95	0.31 ± 0.042	0.025 ± 0.006/8.67 ± 1.92	0.29 ± 0.066
Female	0.07 ± 0.014/18.56 ± 0.64	0.37 ± 0.067	0.031 ± 0.0066/8.27 ± 1.38	0.38 ± 0.10
**Kidney weight, n = 6**				
Male	0.146 ± 0.0048/20.65 ± 0.95	0.71 ± 0.038	0.061 ± 0.0087/8.67 ± 1.92	0.72 ± 0.072
Female	0.121 ± 0.0077/18.56 ± 0.64	0.65 ± 0.03	0.055 ± 0.0074/8.27 ± 1.38	0.67 ± 0.048

Data shown are mean ± SD.

*P < 0.05; ***P < 0.001; as determined by Student’s t-test.

### Malformation of the reproductive system in 4.1N^−/−^ mice

**Figure 3:**
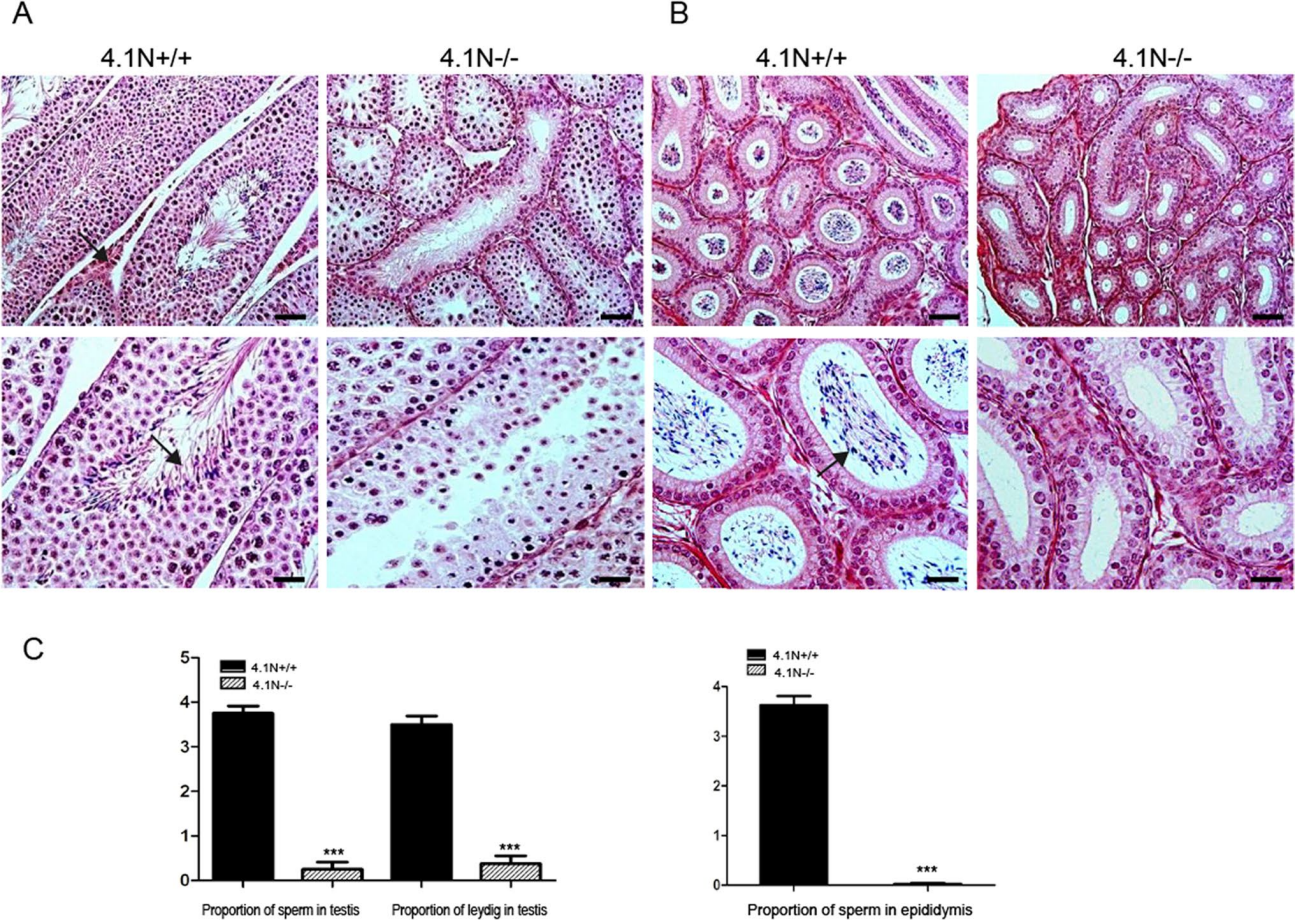
Histopathology of reproductive organs in adult 4.1N^−/−^ male mice. HE-stained sections of 4.1N^+/+^ and 4.1N^−/−^ testis and epididymis (20 ×, upper panels; 40 ×, lower panels). (**A**) Histology showing number of mature sperm in the lumen and normal leydig cells in the interstitium in 4.1N^+/+^ testis (arrow), whereas 4.1N^−/−^ testis didn’t present spermatid and leydig cells. (**B**) Note the abundant sperm in 4.1N^+/+^ epididymis but not in 4.1N^−/−^ epididymis (arrow). The tissues were from 6-weeks old male mice. Scale bar, 100 µm (upper panel) and 50 µm (lower panel). (**C**) Quantification of the proportion of sperm and leydig cells in testis and epididymis. Results are expressed as means ± SD. N = 8, *p ≤ 0.05, **p ≤ 0.01, ***P < 0.001.

Next, histological examination showed hypoplasia of testis in 4.1N^−/−^ male mice with significant changes in mature sperm and in the number of sperm. The seminiferous tubules were reduced in diameter. Spermatogenic cells and spermatocytes were present, but spermatids were not observed. The number of mature spermatozoa was dramatically decreased (Fig. 3C), with few if any detected in seminiferous tubules. No sperm were present in most of the lumens in tubules of the 4.1N^−/−^ testis. The interstitium was sparse, only a few or no leydig cells could be found in 4.1N^−/−^ testis (Fig. 3A). Moreover, no sperm were found in 4.1N^−/−^ epididymis (Fig. 3B). These findings suggest that spermatogenesis was arrested in the spermatocyte stage in the 4.1N^−/−^ mice.

**Figure 4:**
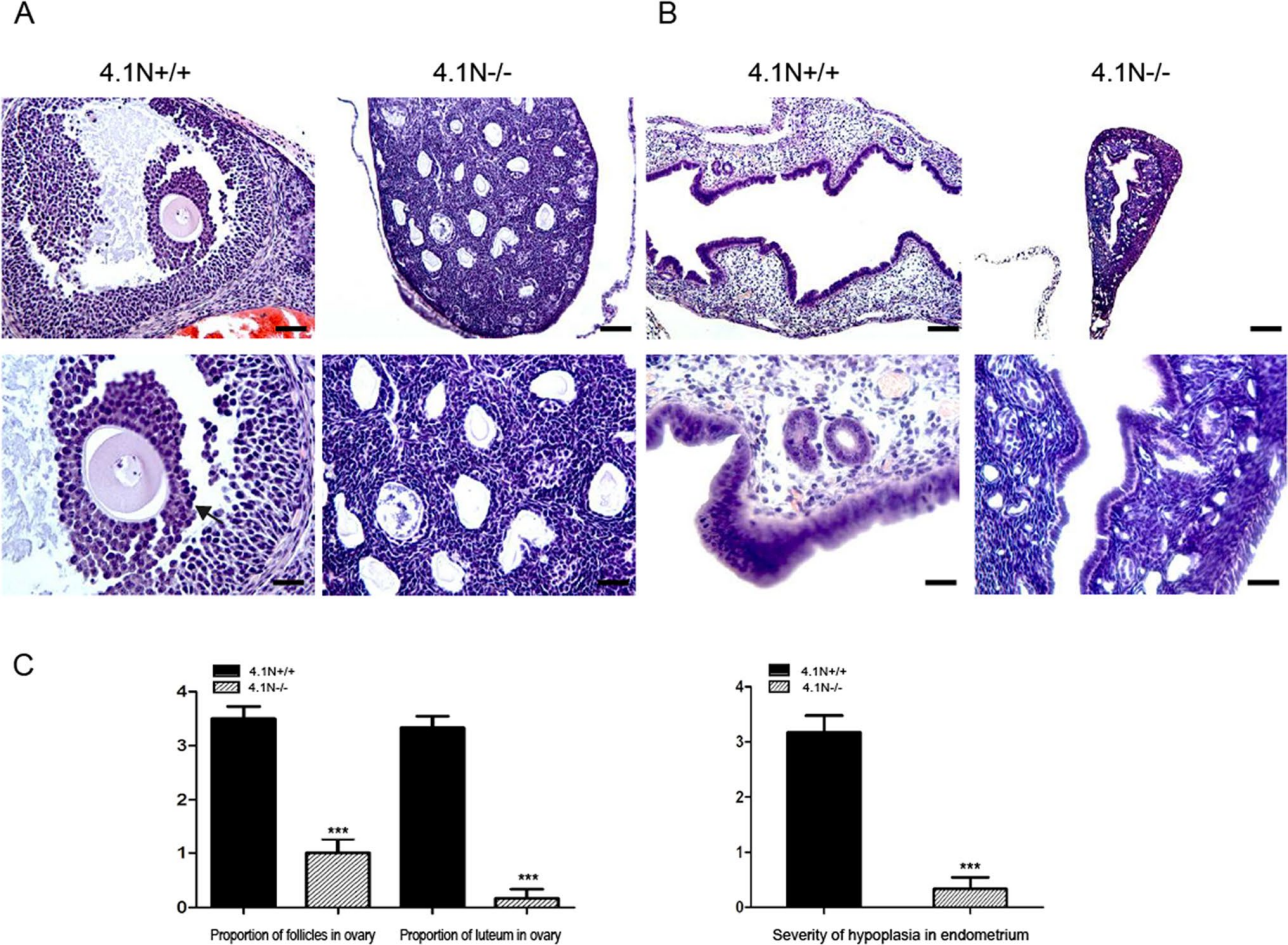
Histopathology of reproductive organs in adult 4.1N^−/−^ female mice. HE-stained sections of 4.1N^+/+^ and 4.1N^−/−^ ovary (upper panel: 20 × , Scale bar, 100 µm; lower panel: 40 × , Scale bar, 50 µm) and uterus (upper panel: 10 × , Scale bar, 200 µm; lower panel: 40 ×, Scale bar, 50 µm). (**A**) A magnified region shows normal antral follicle in wild-type ovary (arrow), but 4.1N^−/−^ ovary shows undeveloped follicles and no corpora lutea was seen in the interstitium. (**B**) A high degree of atrophy was observed in the endometrium in 4.1N^−/−^ uterus compared with 4.1N^+/+^ uterus. 20 ×, upper panels; 40 × lower panels. The tissues were from 6-weeks old female mice. (**C**) Quantification of the proportion of developed follicles and corpora lutea in ovary as well as severity of hypoplasia in the endometrium. Results are expressed as means ± SD. N = 6, *p ≤ 0.05, **p ≤ 0.01, ***P < 0.001.

Hypoplasia was also noted in the ovary and uterus of female 4.1N^−/−^ mice. The follicles of the ovary were largely undeveloped, and their growth arrested in the pre-antral phase of follicle development in 4.1N^−/−^ mice between ages of 7- 23 weeks. No corpora lutea was found, and the interstitium was severely atrophic in 4.1N^−/−^ ovary (Fig. 4A). These findings suggest abnormalities in the maintenance and growth of the follicles. A high degree of hypoplasia was also observed in the endometrium. No estrous cycle changes such as mitotic activity, hyperemic and secretory changes were found in the endometrium (Fig. 4B). In contrast (Fig. 4C), in 4.1N^+/+^ mice of the same age, corpus luteum or corpus albicans of the ovary was evident, and follicles including graafian follicles at different developmental stages were observed. These findings imply that both male and female 4.1N^−/−^ mice exhibit abnormal development of the reproductive system and the immature testis and ovary development account for the infertility of 4.1N^−/−^ mice.

### Other histological findings in 4.1N^−/−^ mice

Meanwhile, a routine microscopic examination of the major organs of 4.1N^–/–^ mice was performed to determine whether 4.1N deficiency might lead to other phenotypes. Histological observation revealed that cells in the zona fasciculata of the adrenal gland had fewer osmiophilic lipid droplets in 4.1N^−/−^ mice compared with 4.1N^+/+^ mice that have significant numbers of lipid droplets in the cytoplasm (Supplementary Fig. S1). However, no changes in adrenal size and weight were noted in 4.1N^−/−^ mice. Microscopic examination of heart, lung, liver, spleen, kidney, pancreas and stomach sections of 4.1N^−/−^ mice did not show noticeable histological alterations in the structure of these organs as compared with 4.1N^+/+^ mice.

### Decreased hormone levels in 4.1N^−/−^ mice

**Figure 5:**
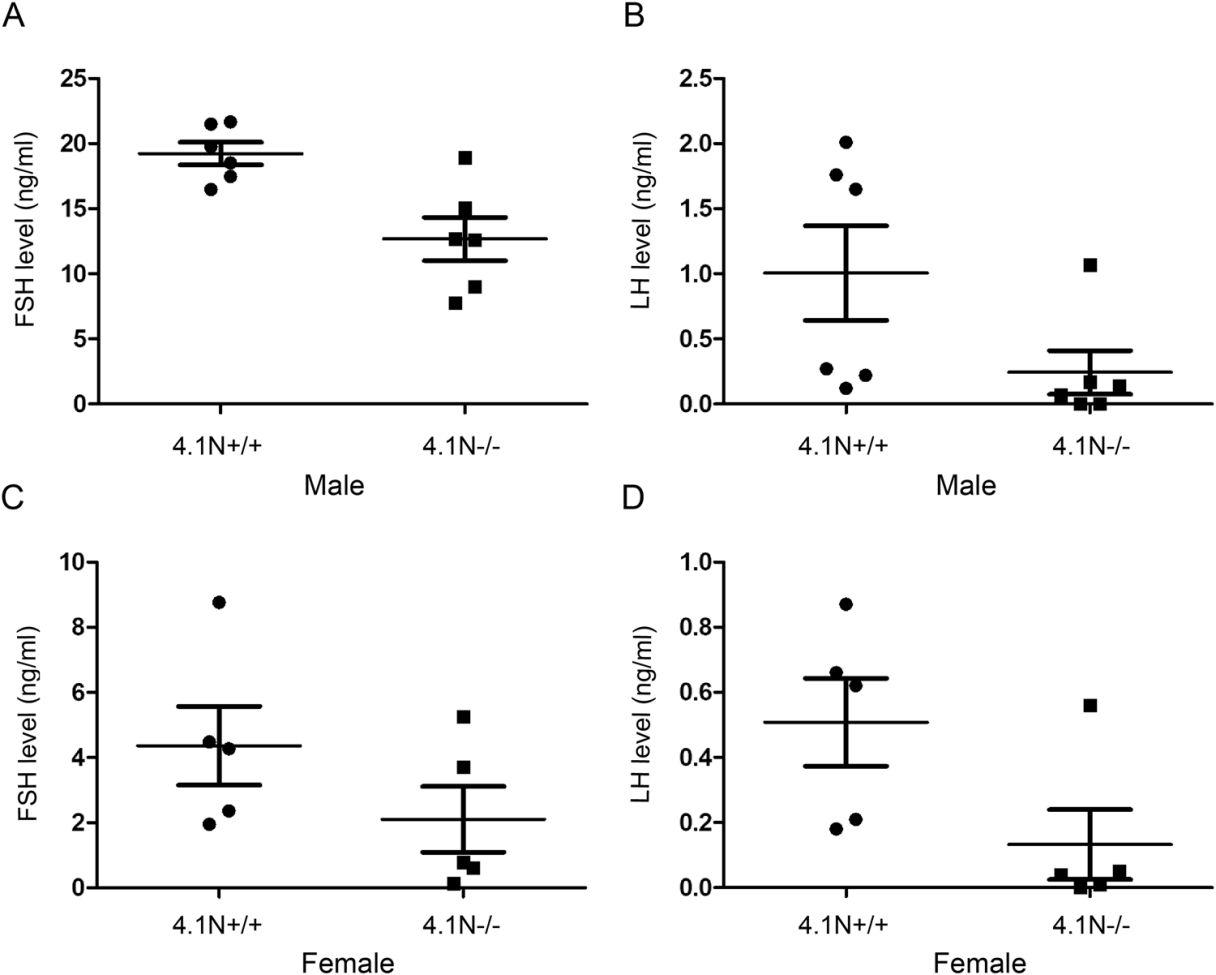
Serum hormone levels in 4.1N^+/+^ and 4.1N^−/−^ mice. The FSH levels in 4.1N^−/−^ male mice were lower than in the age-matched wild-type mice (p = 0.0105, **A**), and LH level showed a modest decreased in 4.1N^−/−^ compared with 4.1N^+/+^ mice (p = 0.0620, **B**). The LH value was decreased in the 4.1N^−/−^ female compared to 4.1N^+/+^ mice (p = 0.0317, **D**), and there was no significant difference for FSH between the two groups (p = 0.222, **C**).

To explore the potential causes for abnormalities in the reproductive system, we measured the serum levels of follicle-stimulating hormone (FSH) and luteinizing hormone (LH) in 4.1N^−/−^ and 4.1N^+/+^ mice (Fig. 5). Analysis of serum hormone concentrations revealed that FSH in 4.1N^−/−^ males was significantly reduced to ~ 66% of that in the age-matched 4.1N^+/+^ mice (p = 0.0105), while this decrease in female knockouts was not significant (p = 0.222). Measurements of LH showed reduced levels in female knockouts compared with wild type (p = 0.0317), while differences in male mice was modest (p = 0.0620) between 4.1N^−/−^ and 4.1N^+/+^ mice (Fig. 5). While growth hormone (GH) levels tended to be decreased in 4.1N^−/−^ compared with 4.1N^+/+^ mice, no statistically significant differences could be documented between the groups (data not shown).

### Changes in hormone expression in 4.1N^−/−^ mice pituitary and hypothalamus

**Figure 6:**
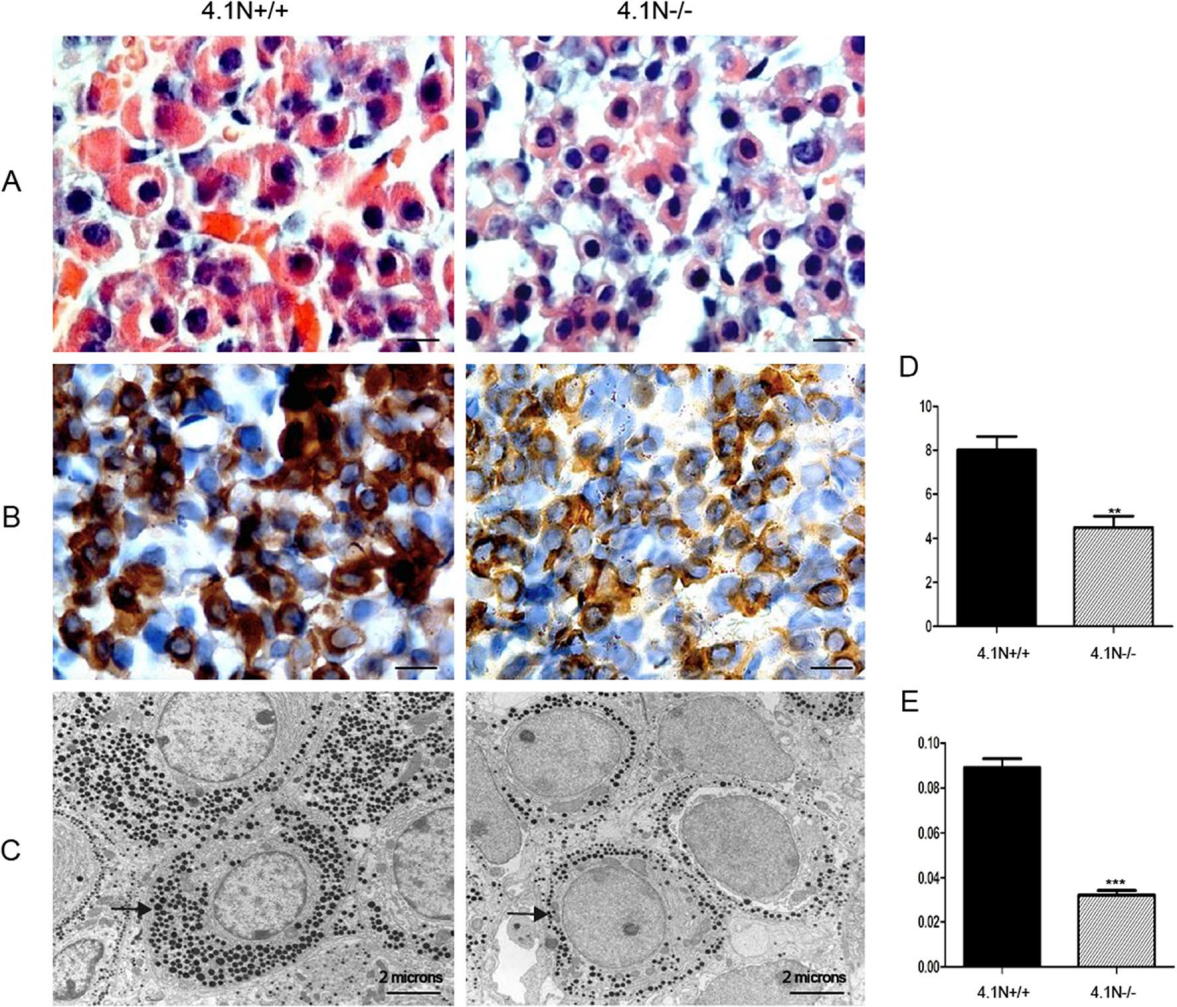
Changes in 4.1N^−/−^ pituitary. **(A)** HE-stained sections showing smaller and less eosin granules in cytoplasm in 4.1N^−/−^ mice pituitary (right100X) compared with 4.1N^+/+^ (left, 100 ×). (**B**) Immunohistochemical staining of GH in the pituitary showing that the expression of GH was decreased in 4.1N^−/−^ mice (right, 100 ×) compared with 4.1N^+/+^ (left, 100 ×). Scale bar, 20 µm. (**C**) Transmission electron microscopic analysis in the pituitary showing that secretory granules (small dark dots) in cytoplasm of hormone-producing cells were dramatically decreased in 4.1N^−/−^ (right, 8680 ×) compared with wild-type (left, 8680 ×), Scale bar, 2 microns. Quantitative analysis for GH immunoreactivity by IHC (**D**) and secretory granules by EM (**E**). Data are shown as means ± SD. N = 12 (6 male, 6 female),*p ≤ 0.05, **p ≤ 0.01, ***P < 0.001.

The pituitary gland and the hypothalamus play a key role in a number of regulatory feedback processes that co-ordinate some physiological phenomena like growth, fertility, metabolism and homeostasis in both genders. To explore the reason for phenotypic changes observed in the 4.1N^−/−^ mice, we examined pituitary tissue from 4.1N^+/+^ and 4.1N^−/−^ mice. The most common cell types of the pituitary pars distalis are growth hormone (GH)-secreting cells. H&E staining show that acidophils are rather diffusely distributed in the secretory cells of normal mice. The basophilic gonadotrophs are responsible for the production of FSH and LH. We noted a decrease in the number and staining intensity of the chromophils, especially acidophils in 4.1N^−/−^ mice. The secretory cells were smaller with fewer eosin granules in cytoplasm in 4.1N^−/−^ mice compared with 4.1N^+/+^ mice (Fig. 6A). The documentation of lower expression of GH in 4.1N^−/−^ pituitary by immunohistochemical staining using specific antibody validated this conclusion (Fig. 6B,D). Electron microscopic examination of pituitary also showed that secretory granules in cytoplasm of the hormone-producing cells were dramatically decreased in 4.1N^−/−^ mice compared with 4.1N^+/+^ mice (Fig. 6C,E). These findings imply that defects in growth and reproductive system development in both male and female 4.1N^−/−^ mice might be the consequence of the downstream effects of neuroendocrine system abnormalities.

**Figure 7:**
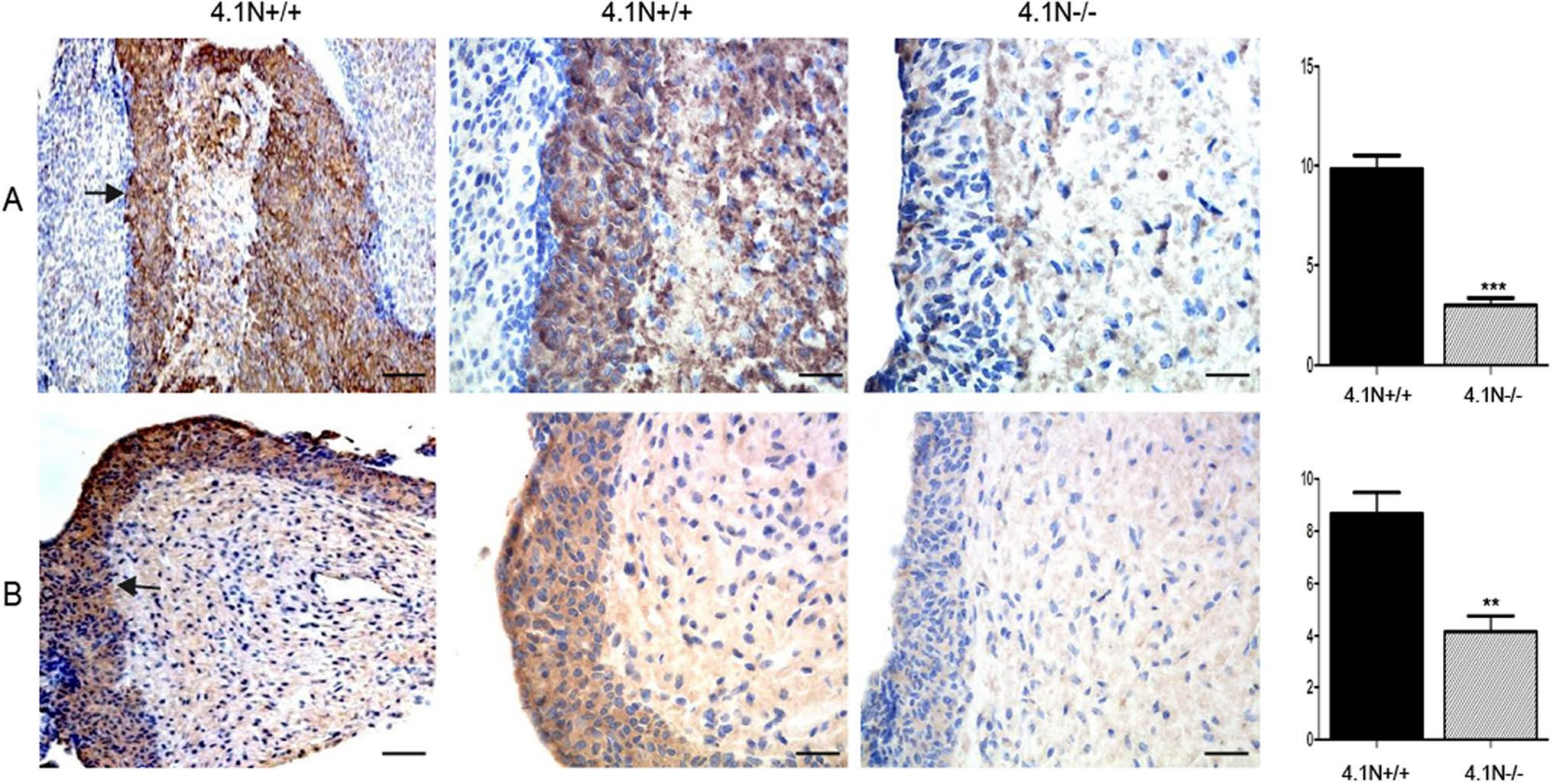
The expression of GnRH and GHRH in 4.1N^+/+^ and 41 N^−/−^ mice pituitary by immunohistochemistry. GnRH (**A**) and GHRH (**B**) immunostaining showing that the two kinds of hormone are mainly expressed in the pars intermedia of mouse pituitary (arrows). The expression of GnRH and GHRH were found dramatically decreased in 4.1N^−/−^ (right panels, 40 × . Scale bar, 50 µm) compared with 4.1N^+/+^ pituitary (left panels, 10 × . Scale bar, 200 µm and middle panels, 40 × . Scale bar, 50 µm). Quantification of immunoreactivity is presented as means ± SD. N = 12 (6 male, 6 female), *p ≤ 0.05, **p ≤ 0.01, ***P < 0.001.

Importantly, expression levels of gonadotropin-releasing hormone (GnRH, Fig. 7A) and growth hormone-releasing hormone (GHRH, Fig. 7B) were significantly reduced in the pituitary of 4.1N^−/−^ mice compared with 4.1N^+/+^ mice. In mouse pituitary, these two releasing hormones are localized in the pars intermedia^18^. The decrease of GnRH and GHRH in pituitary gland from 4.1N^−/−^ mice can in part explain why 4.1N^−/−^ mice have lower FSH and LH and developmental defects in the reproductive system.

**Figure 8:**
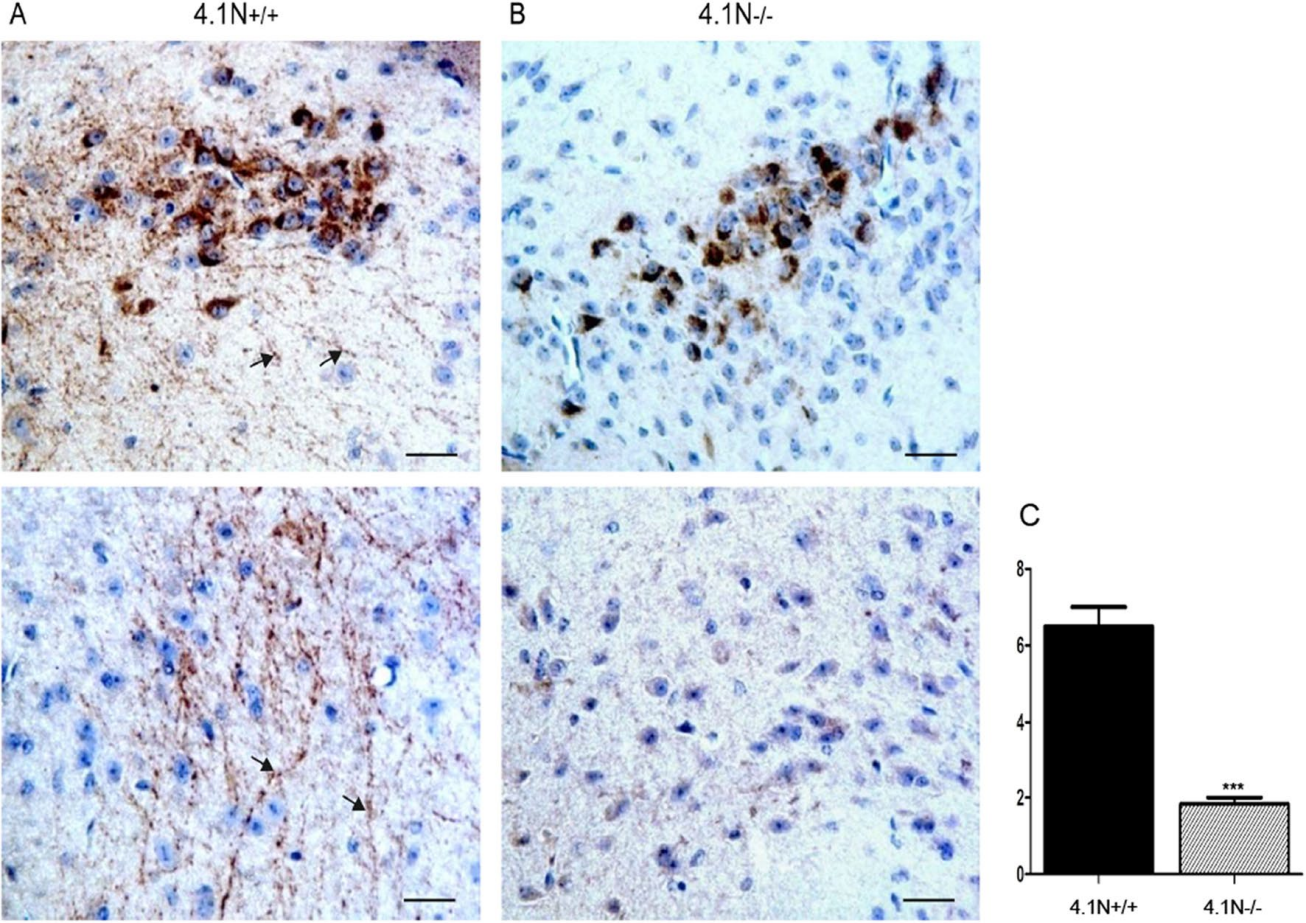
The expression of GnRH in 4.1N^+/+^ and 41 N^−/−^ mice hypothalamus by immunohistochemistry. Immunostaining showing that GnRH-immunoreactive neuronal cell body was present in the hypothalamus region of 4.1N^+/+^ mice (**A**, top panel, 40 ×), the same reactivity was found in the corresponding regions of the 4.1N^−/−^ mice (**B**, top panel, 40 ×). In the 4.1N^−/−^ mice, the axons in the area did not show the expression of GnRH (**B**, bottom panel, 40 ×), in contrast, 4.1N^+/+^ littermates showed much more punctate expression of GnRH in the axons (**A**, bottom panel, arrow, 40 ×). Scale bar, 50 µm. (**C**) Quantification of GnRH immunoreactivity is presented as means ± SD. N = 12 (6 male, 6 female), *p ≤ 0.05, **p ≤ 0.01, ***P < 0.001.

To study the mechanism of decreased expression of releasing hormone observed in the 4.1N^−/−^ pituitary, GnRH immunohistochemical analysis was also performed in the 4.1N^−/−^ and 4.1N^+/+^ hypothalamus (Fig. 8). In contrast to the finding of GnRH immunoreactive signal in both neuronal cell body and axons (punctate) in the median eminence and periventricular area of the third ventricle in the hypothalamus of 4.1N^+/+^ mice, no obvious expression of GnRH was found in the axons of neurons of 4.1N^−/−^ mice, although reactivity was noted in the cell body of GnRH neurons. These results suggest that failure of transfer of GnRH through axons in 4.1N^−/−^ mice.

### Localization of 4.1N protein in mouse pituitary and hypothalamus

Based on the phenotype of 4.1N^−/−^ mice, we also explored the detailed expression pattern of 4.1N in mouse pituitary and hypothalamus by immunohistochemistry and immunogold electron microscopy which has not been previously reported. Immunohistochemistry using the mouse 4.1N antibody revealed several patterns of staining in the hypothalamus area. There was intense staining of cell bodies, dendrites and axons except nucleus and the ependymal cells of the third ventricle. Both diffuse and punctate patterns of staining were found under high magnification (Supplementary Fig. S2).

**Figure 9:**
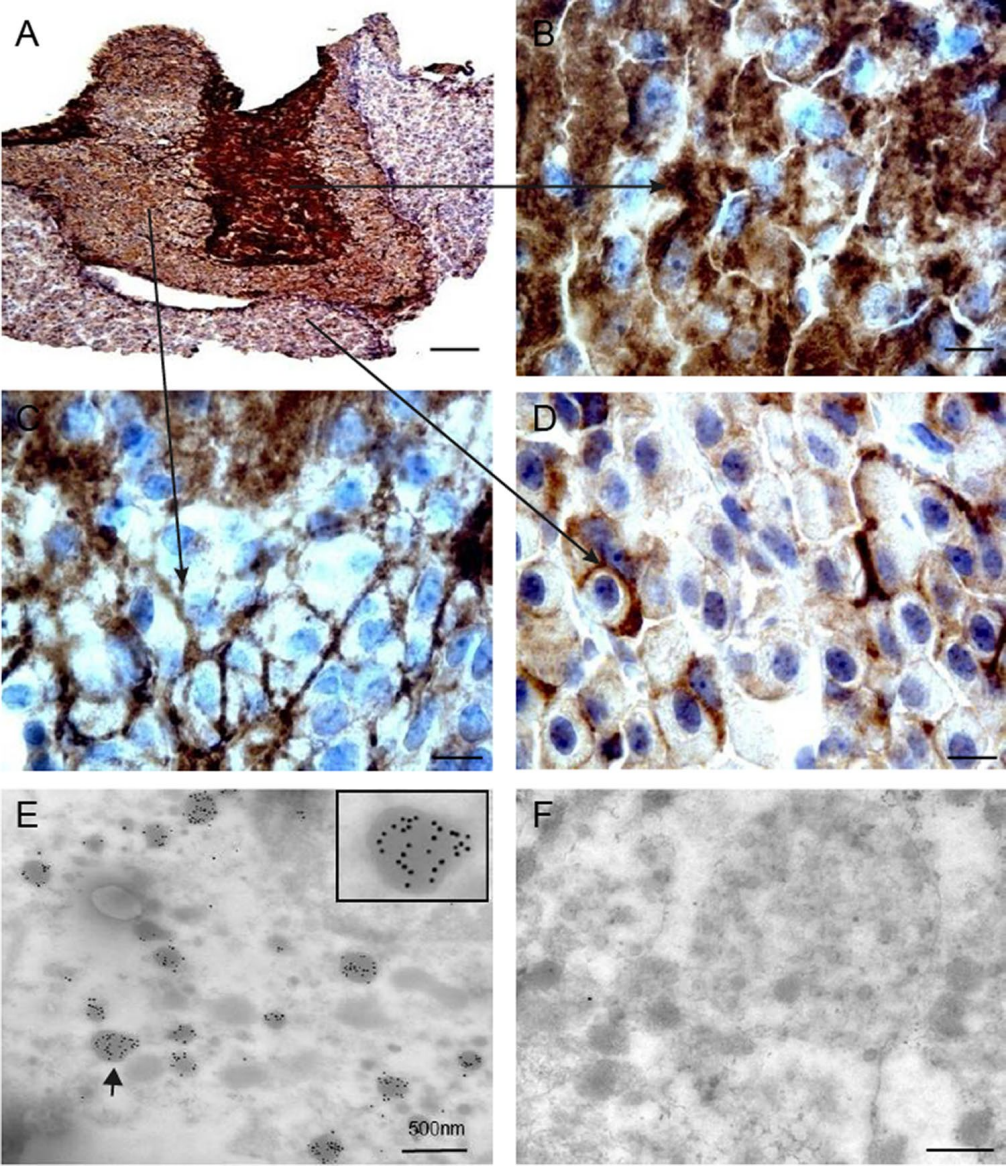
The distribution and subcellular localization of 4.1N in mouse pituitary by immunohistochemistry and immunogold electron microscopy. The expression of 4.1N was found in the pituitary (**A**, 10 × . Scale bar, 200 µm), especially in the neurohypophysis (**B**, 100 ×. Scale bar, 20 µm) and pars intermedia (**C**, 100 ×. Scale bar, 20 µm), which revealed densely speckled and discrete foci pattern. In the pars distalis, punctate labeling that outlined cell bodies in part of cells was observed (**D**, 100 ×. Scale bar, 20 µm). Immunogold electron microscopy shows immunogold labeling for 4.1N in the secretory granules of axons of neurohypophysis and pars intermedia and some cells in adenohypophysis (arrow, and see inset), Scale bar, 500 nm. (**E**). No gold particles were found in a control in which the primary antibody was replaced with nonimmune serum (**F**).

In the pituitary, 4.1N is expressed predominantly in the neurohypophysis and pars intermedia (Fig. 9A). In the neurohypophysis, a densely speckled pattern was observed (Fig. 9B). In the pars intermedia, discrete foci along the axons was noted (Fig. 9C). In pars distalis (adenohypophysis), which contains the secretary cells, punctate labeling that outlined cell bodies was noted in some of the cells (Fig. 9D). Immunogold electron microscopy demonstrated the presence of 4.1N immunoreactivity in the secretory granules in pituitary (Fig. 9E). No gold particles were found in the control in which the primary antibody was replaced with nonimmune serum (Fig. 9F). These findings together with the phenotype of 4.1N^−/−^ mice suggest that 4.1N plays an important role in hormone secretion and transmission along the axonal route.

## Discussion

The findings from the present study have enabled us to document that deficiency of protein 4.1N is associated with abnormal development of the neuroendocrine system leading to phenotypic changes including hypoplasia in the reproductive system and also slow growth. Moreover, the detailed characterization of 4.1N expression in pituitary and hypothalamus enabled the clue of a role for 4.1N in regulating neuroendocrine function.

Although protein 4.1N is enriched in the nervous system^16^, the distribution in neuroendocrine tissue is not clear. We found that high levels of 4.1N were localized to the neurohypophysis and pars intermedia in pituitary and in axons of hypothalamus with speckled or discrete foci pattern. Immunogold electron microscopy confirmed that 4.1N protein mainly exists in secretory granules in cytoplasm. The results suggest that 4.1N participates in hormone secretion or transport in these areas. In 4.1N^−/−^ mice, a remarkable extent of hypoplasia was observed in the reproductive system. The weight of the male and female reproductive organ was noticeably decreased in 4.1N^−/−^ mice, whereas no significant difference for the ratio of organ to body weight was observed for other organs except for a mild decrease in liver. Importantly, histological changes showed underdevelopment of sperm, graafian follicle, uterus and defective of leydig, corpus luteum or corpus albicans. The pathological findings for the testis and ovary as well as growth state led us to explore the upstream changes in 4.1N^−/−^ mice.

It is well established that hormone synthesis, secretion and transport are primarily regulated by the hypothalamic-pituitary–gonadal (HPG) axis. Within the HPG axis, reproductive maturation and function is coordinated by the release of GnRH from a neuronal population in hypothalamus which also consists of other clusters such as GHRH secreting neurons^19–21^. The release of these hypothalamic hormones influences the secretion of anterior pituitary hormones such as FSH, LH and GH that subsequently regulate the function of target organs including testis and ovary as well as in growth^22–26^. However, molecular and mechanistic regulation of hormone transport is yet to be fully defined. The defective sexual development and infertility in the 4.1N^−/−^ mice is likely the result of defects in hormone transport and secretion from the hypothalamohypophysial system.

Our documentation of decreased GnRH and GHRH expression in pituitary pars intermedia in 4.1N^−/−^ mice suggests that the absence of these hormone signals in the pituitary might be attributed to impaired secretion or migration of the releasing hormone granules from the hypothalamic to the pituitary via the hypophyseal-hypothalamic portal system (HHPS). Interestingly, immunohistochemical study confirmed that GnRH are, indeed, absent in the axons of hypothalamus neurons in 4.1N^−/−^ mice. The GnRH immunoreactive signal was present both in neuronal cell body and axons with punctate pattern in the median eminence of the hypothalamus in 4.1N^+/+^ mice. In the 4.1N^−/−^ mice, the expression of GnRH was not detected in the axons, although some reactivity was observed in the cell body of GnRH neurons, indicating that GnRH fails to migrate through axons in 4.1N^−/−^ mice. Furthermore, the decrease of GnRH in pituitary can partly explain why 4.1N^−/−^ mice have lower FSH and LH levels and developmental defects in the reproductive system. These findings suggest that 4.1N is required, directly or indirectly, for the hormone transport and secretion in neuroendocrine system.

It is interesting to note that the extent of changes in FSH and LH levels in 4.1N^−/−^ male and female mice is different. In contrast to the significant decrease in FSH level in 4.1N^−/−^ male mice, the decrease in FSH levels in 4.1N^−/−^ female mice was not statistically significant. This may in part be a reflection of the fact that serum FSH levels are generally lower in female rodents than in males^27^, and there is considerable individual variability in the serum FSH levels masking the difference between the mutant and wild-type mice. The individual variability and stability could account for the finding that the serum GH level showed no statistically significant difference between groups. However, the body weight of 4.1N^−/−^ mice was reduced even in older mice and the expression of GHRH and GH as well as the number of secretory granules were decreased in 4.1N^−/−^ pituitary, suggesting that the slow growth phenotype of 4.1N^−/−^ mice is likely to be of complex etiolgoy.

HPG axis, whose abnormal hormone secretion or transport is associated with clinical feature involving delayed or absent puberty and reproductive dysfunction. Disorder in the development of the GnRH neuroendocrine system leads to absent GnRH secretion or migration, resulting in heterogeneous reproductive disorders such as congenital hypogonadotropic hypogonadism in humans with infertility or decreased fertility^28^. The molecular identities of the complexes responsible for transporting axonal hormone are not well understood. A subclass of semaphorins such as Sema4B and Sema4C characterized by a PDZ-binding motif at their carboxy-terminus that mediates their interaction with the post-synaptic density protein PSD-95/ SAP90, SFAP75/Norbin and SEMCAP-1/GIPC have been implicated in clustering of semaphorins, axon guidance and neurite outgrowth^29–31^.

In line with this pattern, 4.1N co-localizes with the postsynaptic density protein of PSD95 and GluR1 in primary hippocampal cultures and interacts with the GluR1 subunit of the AMPA receptor at excitatory synapses, suggesting that 4.1N may function as a component of the cytoskeletal architecture of excitatory synapses^17,32,33^. GluR1 and GluR3 are synthesized by GnRH neurones in preparation for the enhanced release of GnRH^34^. Our study expands the role of 4.1N to the hypothalamic-pituitary regions, providing further evidence that 4.1N maybe linked to the axonal cytoskeleton which is required to ensure the migration of releasing hormone. It is likely that protein 4.1N plays this role through direct or indirect interactions with other cytoskeletal proteins such as αII- and βII-spectrin which have been shown to contribute to the organization of neurofilaments^35,36^. However, precise interactions in specific axons need to be confirmed in future studies.

In summary, 4.1N^−/−^ mice exhibit unique phenotypes that include low birth rate, slow growth and hypoplasia of reproductive organs due to defects in the neuroendocrine system. Protein 4.1N could be an important component of axons, not only at the hypothalamus, but also in the pituitary region. The presence of 4.1N may be necessary for the transmission of releasing hormone at hypothalamic-pituitary-axon route. The 4.1N knockout mice are likely be a valuable tool for exploring 4.1N function in certain neuroendocrine disorders.

## Methods

### Generation of 4.1N knockout mice

4.1N knockout mice were generated using the similar method for generation of 4.1G knockout mice as described previously^37^. Briefly, embryonic stem (ES) cells, containing a gene trap cassette inserted downstream of exon 1A and upstream of exon 2 in the 4.1N gene, were obtained from the International Gene Trap Consortium (ES cell line FHCRC-GT-S12-6H1). ES cells were microinjected into blastocysts and implanted into recipient female mice to permit development of the embryos into chimeras at the University of California, San Francisco, transgenic facility, and subsequent breeding was performed to select founders with germ line transmission of the gene trap allele. The mice were initially bred and screened at the Lawrence Berkeley National Laboratory (LBNL) animal facility and then maintained at the animal facility of New York Blood Center under specific-pathogen-free conditions. All animal experiments and protocols were reviewed and approved by Institutional Animal Care and Use Committees from both the New York Blood Center and Lawrence Berkeley National Laboratory, and conducted in accordance with the New York Blood Center and Lawrence Berkeley National Laboratory guidelines and regulations. Mice were backcrossed more than 9 generations into a C57BL/6 background, and, in all experiments, comparisons were made between littermate wild-type (4.1N^+/+^) and homozygous mice (4.1N^−/−^). The genotyping was routinely performed by PCR as described below. In total, we examined forty-four 4.1N^−/−^ (21 males and 23 females) and the same number of wild-type and heterozygous mice. The mice were mainly divided into two groups: 3–5 weeks old and 6–9 weeks old. A few mice older than 9 weeks were also analyzed.

### Genotyping

Genomic DNA was extracted from mouse-tails. *Genotyping* was performed by multiplex PCR to identify mice inheriting the targeted 4.1N gene and to distinguish wild type and heterozygous mice from homozygous. The wild type 4.1N allele was specifically amplified using primers 4.1N-F1: 5′-TTGAGTTCCAGGACAGCCAGGGTTAC-3′ and 4.1N reverse: 5′-AGAGAGCCTTGAAAGAGACCAGACGAG-3′), while the mutant allele carrying the gene trap insertion was detected using primers 4.1N-F2 (5′-GTGATGAACTGATGGAAGGATAGCC-3′) and β-geo-Rev (5′-AGTATCGGCCTCAGGAAGATCGCAC-3′).

### Autopsy and histopathological examination

Mice were anesthetized with pentobarbital and perfused through the heart with PBS (pH7.4) containing 4% paraformaldehyde (PFA). After sacrificing the mice, various tissues or organs were rapidly dissected and fixed in 10% phosphate-buffered formalin solution. The testis were fixed in Bouin’s solution. The tissues were subsequently embedded in paraffin, and serial sections (4-μm thick) were cut and mounted on slides. The sections were stained with hematoxylin–eosin (HE). Immunohistochemistry was performed using various antibodies. The stained sections were examined microscopically. The number of testis and ovary analyzed were from 8 male and 6 female, separately. The other tissues were from 6 male and 6 female. Histopathology was assessed independently by two pathologists who were blinded to the different groups. Microscopic semiquantitative scoring was calculated on a scale of 0 to 4 as follows: 0 (no sperm, leydig cells, follicles or corpus luteum), 1 (minimal, < 10%), 2 (mild, 10 to 30%), 3 (moderate, 31 to 65%), and 4 (severe, > 65%).

### Generation of anti-4.1N antibody

Polyclonal antibody against 4.1N was raised in rabbit using His-tagged recombinant N-terminal headpiece of 4.1N as antigen. The sequence of the peptide is unique to 4.1N and does not share any homology with 4.1R, 4.1G or 4.1B. The antibody was affinity purified on Sulfolink Coupling Gel (Pierce Biotechnology, Inc.). Antibody specificity was examined by Western blotting using recombinant 4.1R, 4.1G, 4.1N and 4.1B. As demonstrated in our previous published study, the 4.1N antibody was highly specific for 4.1N and did not recognize other members of the protein 4.1 family^38^.

### Western blot

Western blot was performed as described previously^6^. Briefly, tissues from adult 4.1N^+/+^ and 4.1N^−/−^ were homogenized by sonication in ice-cold lysis buffer (0.32 M sucrose, 0.01 M HEPES, pH 7.4, 2 mM EDTA, 1 mM DTT, protease inhibitor cocktail) in 1.5 ml tubes. After centrifugation at 900×*g* for 5 min, the supernatant was collected and the protein concentration measured by Bradford method using bovine serum albumin as standard. 30 μg of proteins were run on 8% Tris–glycine gels and subsequently transferred onto nitrocellulose membrane (BIO-RAD, Hercules, CA). Membranes were blocked for 1 h at room temperature in blocking buffer (PBS + 0.1% Tween-20 + 4% nonfat dry milk + 1% BSA) followed by overnight incubation at 4 °C with primary 4.1N antibody (1: 2000 dilution) and rabbit polyclonal anti-GAPDH antibody (1: 5000 dilution) in blocking buffer. After washes with 0.1% PBST, membranes were incubated for 1 h at room temperature with rabbit HRP-conjugated secondary antibody (Jackson ImmunoResearch Laboratories, Inc, West Grove, PA) diluted 1:5000 in blocking buffer. Following extensive washing, membranes were processed using chemiluminescent reagent (Thermo Scientific, Rockford, IL).

### Immunohistochemistry

Immunohistochemical analysis was performed as described previously^6^. Briefly, 4.0 μm thick sections were de-paraffinized in xylene and dehydrated using graded ethanol solutions. Endogenous peroxidase activity was quenched with peroxidase blocking reagent for 6 min. The tissue sections were pretreated with target retrieval solutions of high pH (for 4.1N immunostaining) or with low pH (for GH-growth hormone, GnRH-gonadotropin releasing hormone and GHRH-growth releasing hormone immunostaining) and heated in microwave for 15 min. Following three washes with PBS, the sections were incubated with specific antibodies, at a dilution of 1:100 for anti-4.1NHP, 1:50 for anti-GH(R&D systems, BAF1067), 1:50 for anti-GnRH (Thermo, PA1-121)^39^, 1:200 for anti-GHRH(Abcam, Ab48617), overnight at 4 °C. After several washes in PBS, the sections were incubated with HRP-conjugated secondary antibody (Dako North America. Inc. Carpinteria, CA, USA) for one hour at room temperature followed by exposure to the chromogen (3,3′ diamino benzidine tetrachloride, DAB) system for 1–5 min. Slides were counterstained with haematoxylin and examined by light microscopy (Leica DM 2000, Leica Microsystems Inc.). A semiquantitative scoring system was used. The intensity of immunoreactivity was measured on a 0–3 scale. A score of 0 indicated no reactivity, a score of 3 denoted an intensity of cell reactivity with dark brown. The percentage positivity was based on as follows: A score of 0 indicated no cell immunopositivity, 1, 1–10% positive cells, 2, 11–40% positive cells, 3, 41–70% positive cells and 4, > 70% positive cells. The final scores for intensity and percentage of immunopositivity for each antibody were multiplied to give a number from 0 to 12.

### Electron microscopy and immunogold labelling

Pituitary samples were fixed with 3% glutaraldehyde in 0.1 M sodium cacodylate buffer, pH 7.4, for 2 h at room temperature and rinsed in the same buffer several times. Following post-fixation in 1% osmium tetroxide solution, the samples were dehydrated with increasing concentrations of ethanol and small pieces of the tissue were embedded in EMBed-812 media (Electron Microscopy Sciences, Fort Washington, PA) and cured at 60 °C for 24 h. Ultra-thin sections (65–70 nm) were cut with an MT-XL ultra-microtome (RMC Products, USA), and stained with a Uranyl acetate solution followed by Reynold’s Lead Citrate Stain solution. Sections were imaged using a Tecnai G2 Spirit BioTWIN Transmission Electron Microscope (FEI Company, Holland). The area ratio of secretory granules in cytoplasm were calculated with QWin image analysis software (Germany).

For immunogold labeling**,** tissue specimens, ~ 1 mm^3^ in size, were collected and fixed in the mixture of 4% paraformaldehyde and 0.1% glutaraldehyde in 0.1 mol/L cacodylate buffer, pH 7.4, for 1 h at 4 °C. After washing with 0.1 M cacodylate buffer and treatment with 50 mM ammonium chloride to quench residual aldehydes, the fixed tissue was dehydrated and embedded in LR-White Resin (Electron Microscopy Sciences, Hatfield, PA). Ultrathin sections of embedded tissue were cut with a diamond knife and mounted on to parlodion coated nickel grids. Non-specific binding was blocked by applying nonimmune serum to the sections, followed by incubation overnight at 4 °C with 4.1N primary antibody diluted 1:10 in 0.05 mol/L Tris-buffered saline (TBS), supplemented with 0.1 mol/L bovine serum albumin (BSA). After thorough rinsing with TBS containing 0.1% BSA and 0.5% Tween 20, the sections were incubated with 15-nm gold-conjugated goat-anti-rabbit IgG (Amersham Biosciences) diluted 1:20 in TBS for 2 h at room temperature. Following thorough rinsing with TBS, the sections were treated with 4% aqueous uranyl acetate to enhance contrast and imaged using the transmission electron microscope. For control, either the primary antibody was omitted or nonimmune serum was used.

### Hormone assays

Mice serum were collected and sent to Laboratory of Comparative Pathology, Memorial Sloan Kettering Cancer Center and Weill Medical College at Cornell University for hormone assay. FSH and LH levels were measured by the radio immunoassay and GH levels by ELISA enzyme immunoassay.

### Statistical analysis

All numerical data were presented as mean ± SD. Statistical analysis were performed by Student’s t-test and one-way ANOVA, values of P < 0.05 were considered to be significant.

## Supplementary information

Supplementary Figures.

## Data Availability

The data generated and/or analysed during the current study are available from the corresponding authors on reasonable request.
