# Supplementary material for: Selective effects of protein 4.1N deficiency on neuroendocrine and reproductive systems
Source: Sci Rep. 2020 Oct 12;10:16947. doi: 10.1038/s41598-020-73795-6 (PMC7550591; doi:10.1038/s41598-020-73795-6)
Supplement: Supplementary file 1 — Supplementary Figures. [file 41598_2020_73795_MOESM1_ESM.pdf]

Selective effects of protein 4.1N deficiency on neuroendocrine and reproductive systems

Hua Wang[1,2], Marilyn Parra[3], John G Conboy[3], Christopher D. Hillyer[1], Narla Mohandas[1] and Xiuli An[4*]

[1]Red Cell Physiology Laboratory, New York Blood Center, New York, NY 10065, USA

[2] Department of Pathology, School of Basic Medical Sciences, Peking University Health Science Center, and Peking University Third Hospital, Beijing 100191, China

[3]Biological Systems and Engineering Division, Lawrence Berkeley National Laboratory, Berkeley, CA 94720, USA

[4]Laboratory of Membrane Biology, New York Blood Center, New York, NY 10065, USA

Correspondence and requests for materials should be addressed to Xiuli An, Laboratory of Membrane Biology, 310 East 67th St, New York, NY 10065, USA. Tel: 212-570-3247, email: xan@nybc.org

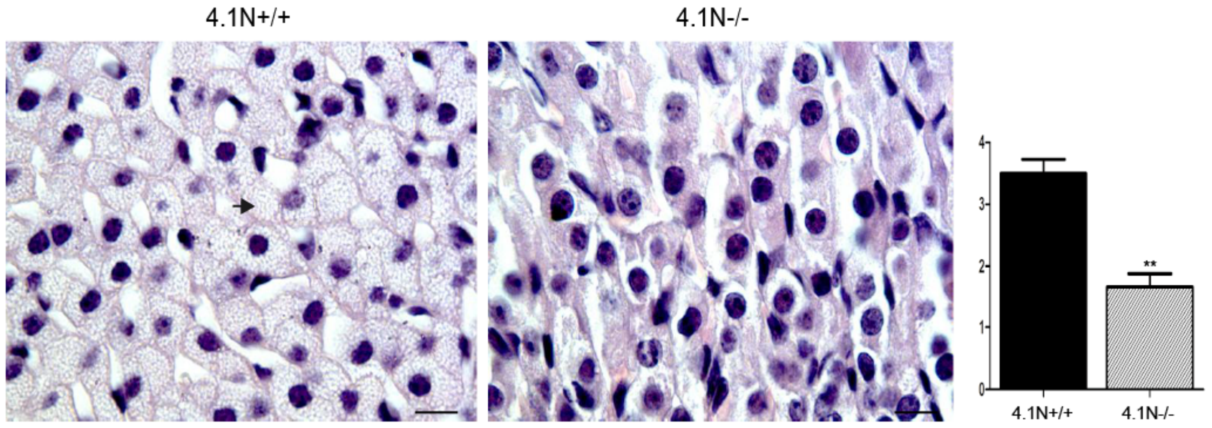

Supplementary Fig.1 Differences between 4.1N$^{+/+}$ and 4.1N$^{-/-}$ adrenal gland. HE staining revealed that cells in the zona fasciculata of adrenal gland had fewer osmiophilic lipid droplets in 4.1N$^{-/-}$ mice compared with wild-type mice (arrow,100×. Scale bar, 20μm). Quantification of proportion of lipid droplets in the adrenal gland are expressed as means ± SD. N=12 (6 male, 6 female), *p ≤0.05, **p ≤ 0.01, ***P< 0.001.

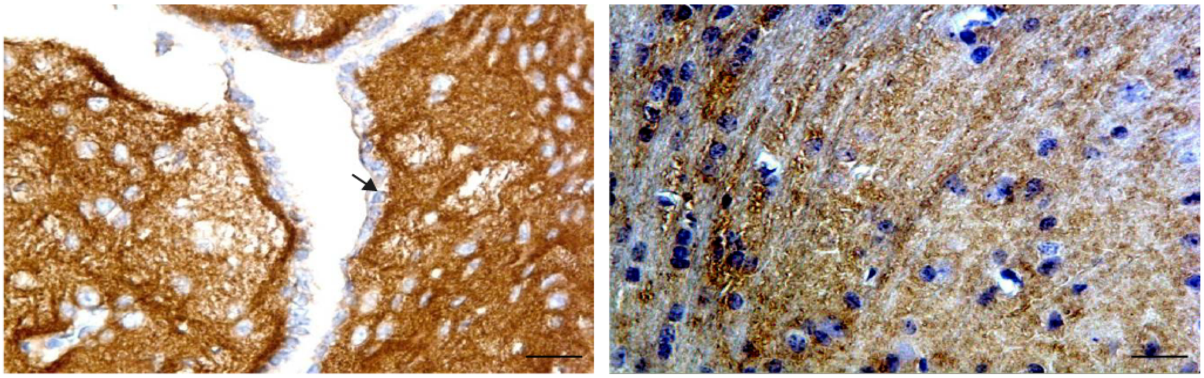

Supplementary Fig.2 Expression of 4.1N in the hypothalamus. 4.1N immunohistochemical staining revealed diffuse expression in the hypothalamus area, including cell bodies and axons except for the nucleus and the ependymal cells of the third ventricle (arrow, 40×. Scale bar, 50μm).

A

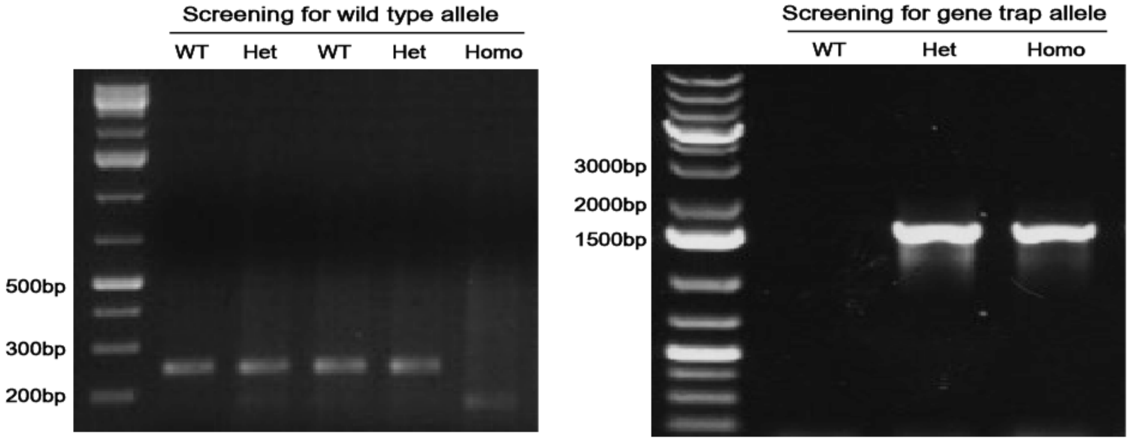
Screening for wild type allele
WT
Het
WT
Het
Homo
500bp
300bp
200bp
Screening for gene trap allele
WT
Het
Homo
3000bp
2000bp
1500bp

B

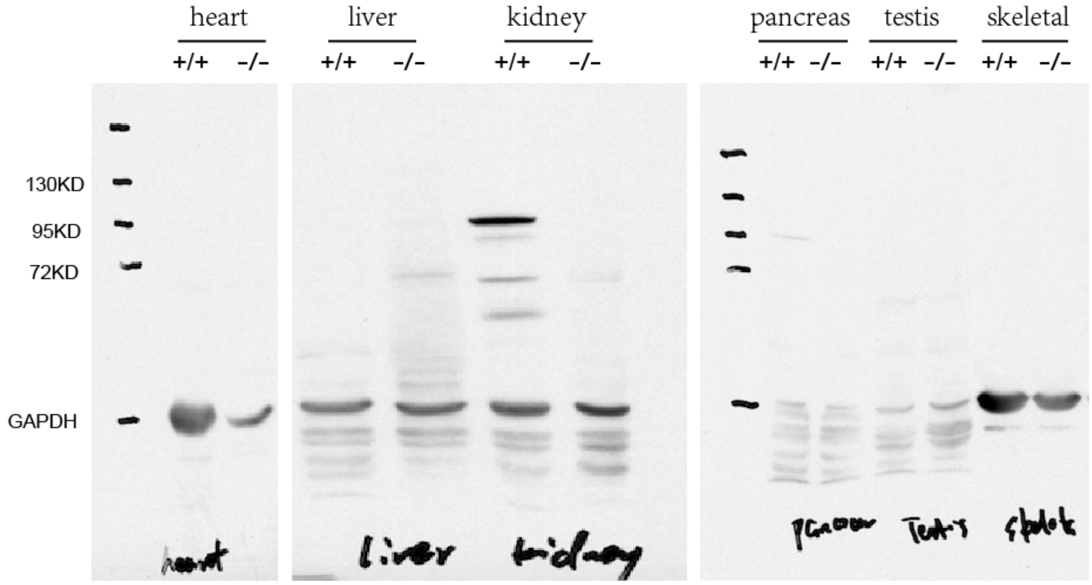
heart
liver
kidney
pancreas
testis
skeletal
+/+ -/-
+/+ -/-
+/+ -/-
+/+ -/-
+/+ -/-
+/+ -/-
130KD
95KD
72KD
GAPDH

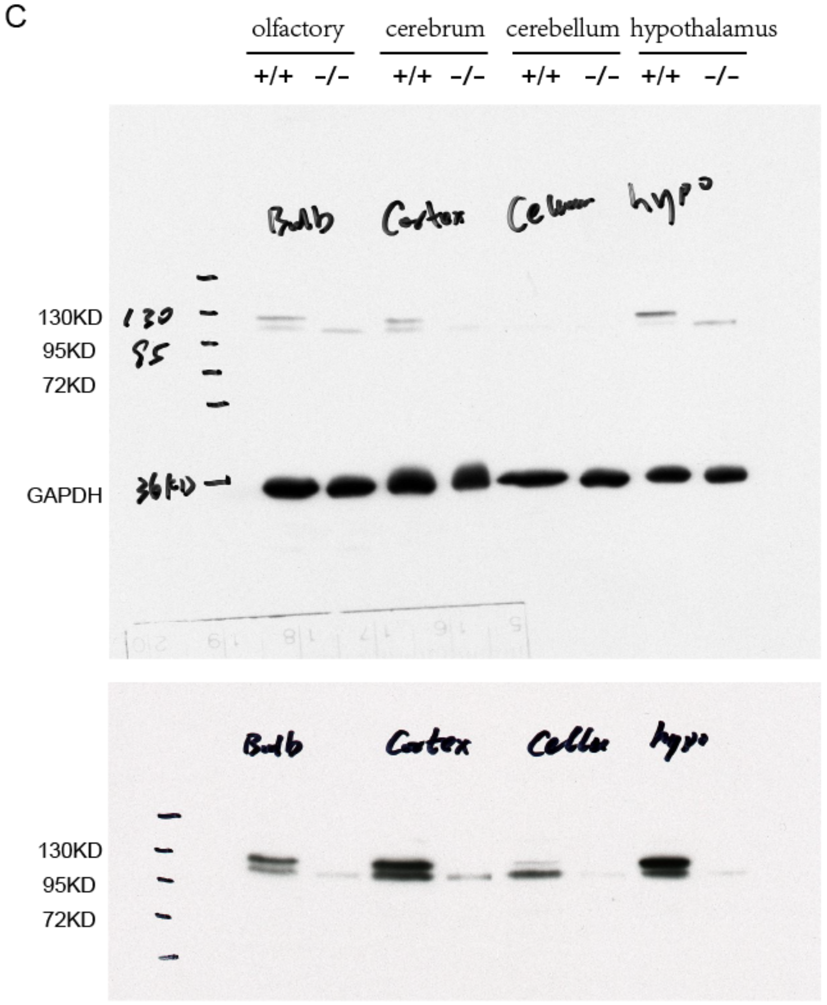

Supplementary Fig.3 (A) Genotype analysis by PCR. (B) Western blotting analyses of multiple tissues. The membrane was probed with both anti-4.1N and anti-GAPDH antibody at the same time. (C) Western blot analyses of various regions of brain. Upper panel: the membrane was probed with both anti-4.1N and anti-GAPDH antibody at the same time; Lower panel: the membrane was probed with anti-4.1N antibody only.

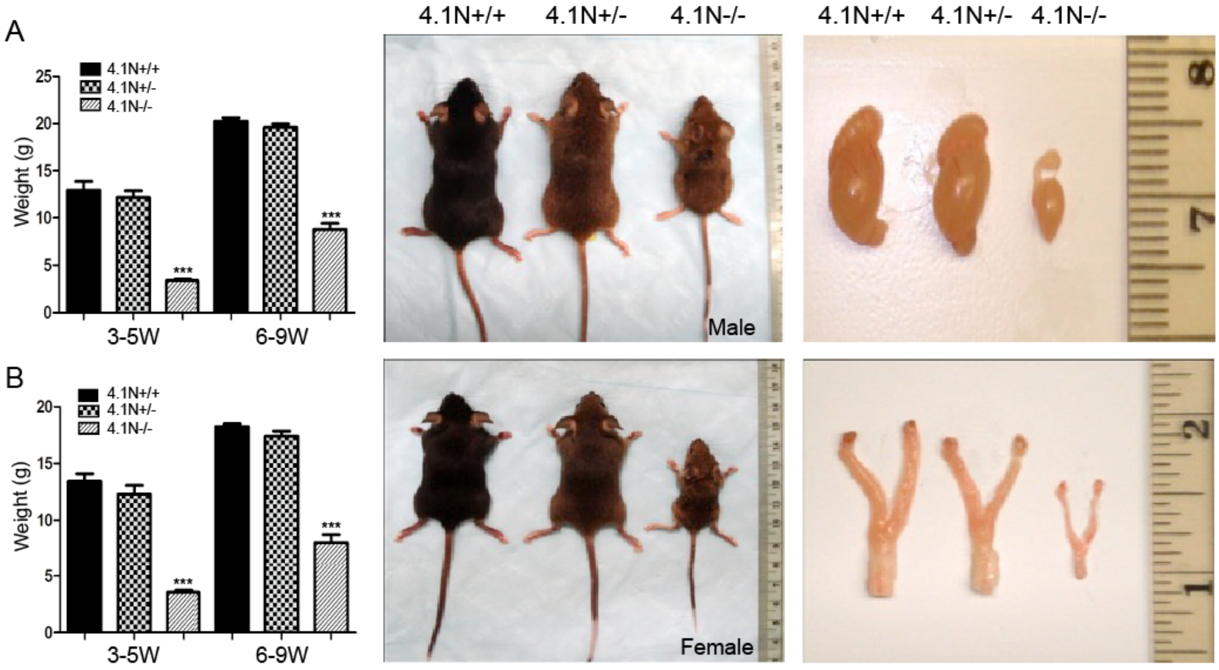

Supplementary Fig.4 Outward appearance and gross appearance of the reproductive organs of adult mice. 4.1N$^{-/-}$ 8-week-old male (A) and 6-week-old female (B) mice showed obvious growth retardation associated with decreased weight and size compared to 4.1N$^{+/+}$ littermates, while no significant differences were observed between WT and 4.1N+/- mice. Bar graphs demonstrating body weight of 4.1N-null mice was reduced compared with wild-type and heterozygous littermates both in males (A) and females (B) at 3-5 weeks (P<0.001, n=6/each group) and 6-9 weeks (P<0.001, n=10/each group). Macroscopic view of the testis and epididymis at 8 weeks of age from the control 4.1N$^{+/+}$ littermates and 4.1N$^{-/-}$ mice showed that the male reproductive organs are dramatically smaller in the 4.1N$^{-/-}$ mice (A). Bottom panels are macroscopic views of the ovary and uterus at 20 weeks of age from the control 4.1N$^{+/+}$ littermates (left) and 4.1N$^{-/-}$ mice showing these organs are much smaller in the 4.1N$^{-/-}$ mice. There is no significant differences between WT and 4.1N+/- mice.

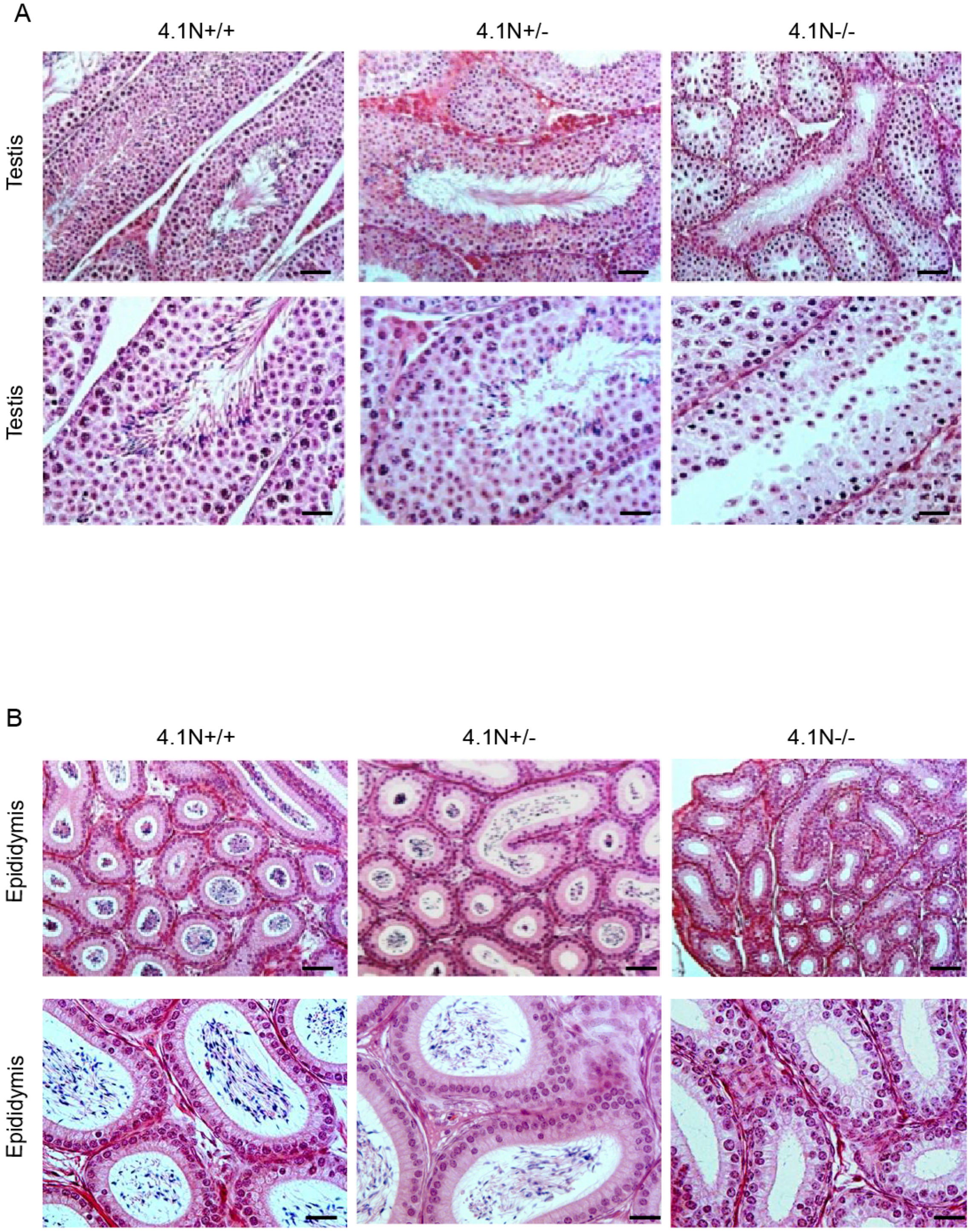
A
4.1N+/+
4.1N+/-
4.1N-/-
Testis
Testis
B
4.1N+/+
4.1N+/-
4.1N-/-
Epididymis
Epididymis

C

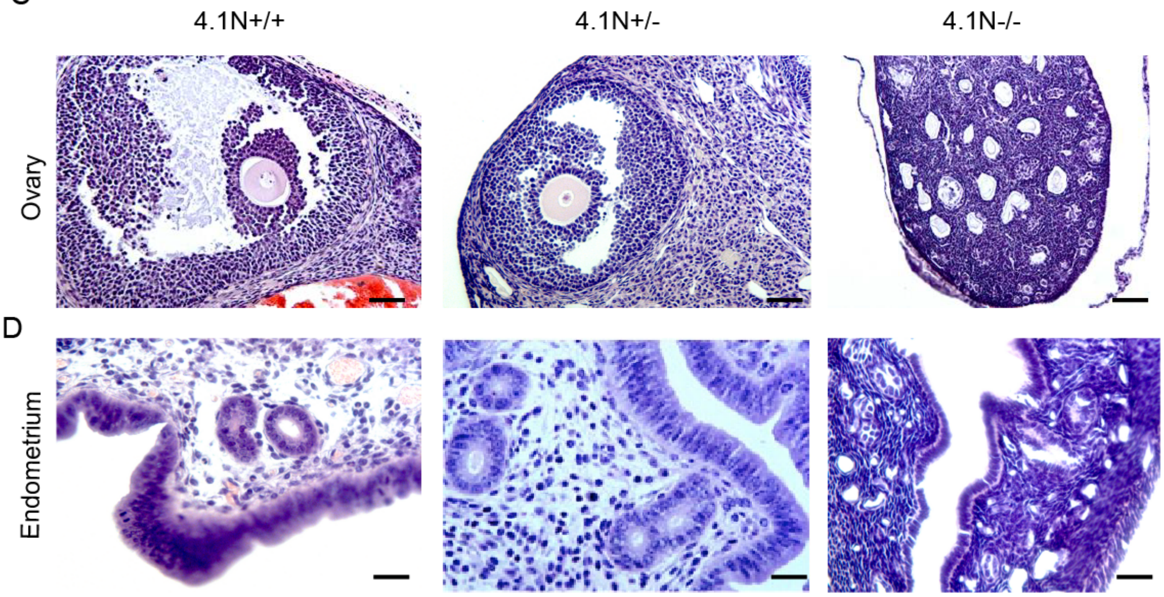
4.1N+/+
4.1N+/-
4.1N-/-
Ovary
Endometrium

D

E

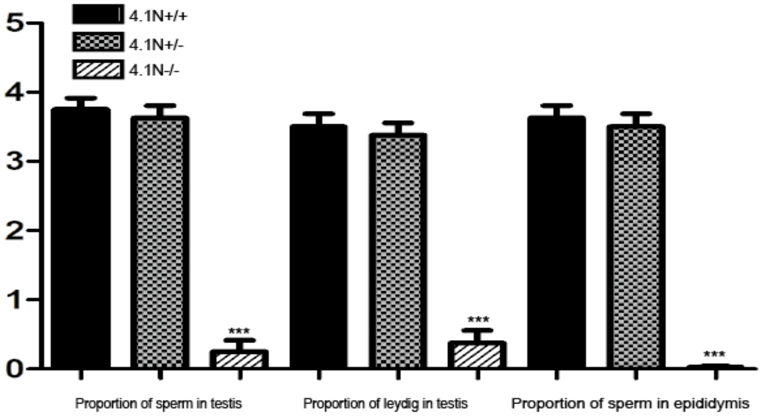
4.1N+/+
4.1N+/-
4.1N-/-
5
4
3
2
1
0
***
***
***
Proportion of sperm in testis
Proportion of leydig in testis
Proportion of sperm in epididymis

F

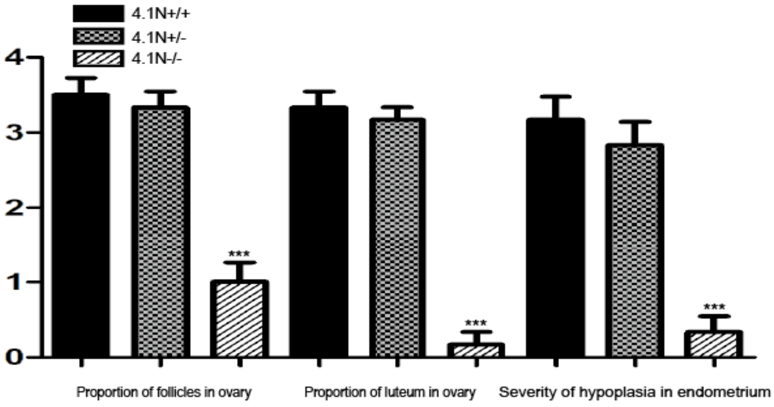
4.1N+/+
4.1N+/-
4.1N-/-
4
3
2
1
0
***
***
***
Proportion of follicles in ovary
Proportion of luteum in ovary
Severity of hypoplasia in endometrium

Supplementary Fig.5 Histopathology of reproductive organs in 6-weeks old 4.1N$^{+/+}$, 4.1N$^{+/-}$, 4.1N$^{-/-}$ male and female mice. HE-stained sections of testis [A,Scale bar, 100µm (upper panel) and 50 µm (lower panel)], epididymis [B, Scale bar,100µm (upper panel) and 50 µm (lower panel)], ovary (C, Scale bar,100µm) and endometrium (D, Scale bar,50µm). Histology showing number of mature sperm in the lumen and normal leydig cells in the interstitium in 4.1N$^{+/+}$ and 4.1N$^{+/-}$ testis and epididymis, whereas 4.1N$^{-/-}$ testis didn't present spermatid and leydig cells. (C) A magnified region shows normal antral follicle in 4.1N$^{+/+}$ and 4.1N$^{+/-}$ ovary, but 4.1N$^{-/-}$ ovary shows undeveloped follicles and no corpora lutea was seen in the interstitium. (D) A high degree of atrophy was observed in the endometrium in 4.1N$^{-/-}$ uterus compared with 4.1N$^{+/+}$ and 4.1N$^{+/-}$ uterus. 20×, upper panels, 40× lower panels. (E) Quantification of the proportion of sperm and leydig cells in testis and epididymis. N=8. (F) Quantification of the proportion of developed follicles and corpora lutea in ovary as well as severity of hypoplasia in the endometrium. Results are expressed as means ± SD. N=6, *p ≤0.05, **p ≤ 0.01, ***P< 0.001.

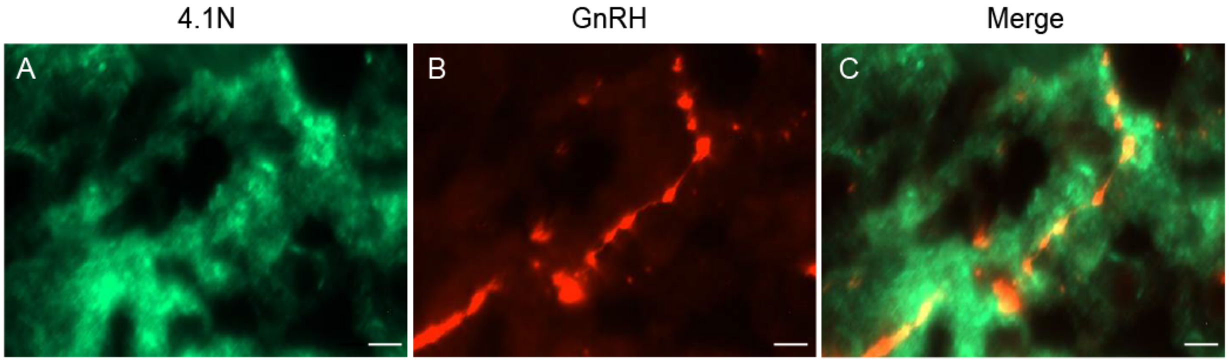

Supplementary Fig.6 Double staining of 4.1N and GnRH in hypothalamus. 4.1N (green) colocalizes in certain regions with GnRH (red) in the axons of hypothalamus. 40×. Scale bar, 50µm.
